# Constructing ROS-Responsive Supramolecular Gel with Innate Antibacterial Properties

**DOI:** 10.3390/pharmaceutics15082161

**Published:** 2023-08-19

**Authors:** Fen Zheng, Wei Du, Minggang Yang, Kaige Liu, Shanming Zhang, Long Xu, Yong Wen

**Affiliations:** 1 School of Materials Science and Chemical Engineering, Ningbo University, Ningbo 315211, China; 2 Department of Urology, Zhujiang Hospital, Southern Medical University, Guangzhou 510515, China; 3 Research Center for Human Tissues and Organs Degeneration, Shenzhen Institute of Advanced Technology, Chinese Academy of Sciences, Shenzhen 518055, China

**Keywords:** antibacterial gel, supramolecular gel, ROS-responsive, low molecular weight gelator, biocompatibility

## Abstract

Bacterial infections, especially antibiotic-resistant bacterial infections, pose a significant threat to human health. Supramolecular gel with innate antibacterial properties is an advanced material for the treatment of bacterial infections, which have attracted great attention. Herein, a reactive oxygen species (ROS)-responsive innate antibacterial supramolecular gel is developed by a bottom-up approach based on phenylalanine and hydrazide with innate antibacterial properties. The structure of gelators and intermediate products was characterized by proton nuclear magnetic resonance (^1^H NMR) and a high-resolution mass spectrum (HRMS). The results of ^1^H NMR and the Fourier transform infrared spectrum (FT–IR) experiment disclosed that hydrogen bonding and the π–π stacking force are the important self-assembly driving forces of gelators. The microstructure and mechanical properties of gel were studied by Scanning electron microscope (SEM) and Rheometer, respectively. An in vitro degradation experiment proved that the gelator has ROS-responsive degradation properties. The in vitro drug release experiment further manifested that antibiotic-loaded gel has ROS-responsive drug-release performances. An in vitro cytotoxicity experiment showed that the supramolecular gel has good biocompatibility and could promote cell proliferation. The in vitro antibacterial experiment proved that the supramolecular gel has excellent inherent antibacterial properties, and the antibacterial rate against *Staphylococcus aureus* (*S. aureus*) and *Escherichia coli* (*E. coli*) was 98.6% and 99.1%, respectively. The ROS-responsive supramolecular gel as a novel antibacterial agent has great application prospects in treating antibiotic-resistant bacterial-infected wounds and preventing the development of bacterial resistance.

## 1. Introduction

As the first barrier of the human body, skin plays an important role in protecting body tissues and preventing bacterial invasion. Human beings are vulnerable in daily life. Once injured, defects and wounds may appear on the skin. If the wound is exposed to air and not treated promptly, wounds are susceptible to bacterial infection. Bacterial infections result in delayed wound healing, severe suffering, and associated secondary health problems [1]. Bacterial infections pose a significant threat to human health, and severe bacterial infections can lead to nonhealing wounds, sepsis, or death [2]. According to statistics, bacterial infections cause approximately 15-million deaths annually [3]. Antibiotics were widely and traditionally used for treating bacterial infections. Unfortunately, due to the abuse of antibiotics and the evolution of bacteria, an increasing number of antibiotic-resistant bacteria have emerged. Therefore, there is an urgent need to develop new antibiotics or antibacterial materials to treat wounds infected with antibiotic-resistant bacterial.

More researchers are committed to developing new antibacterial materials, such as antimicrobial peptides, graphene, metal–organic frameworks (MOFs), metal ions and nanoparticles, and antimicrobial hydrogel, for the treatment of antibiotic-resistant bacterial infections [4,5,6,7,8]. Among these antibacterial materials, antibacterial hydrogel is one of the most promising antibacterial materials; this has aroused great research interest of researchers. Antibacterial hydrogel as a wound dressing has especially significant advantages in promoting the healing of bacterial-infected wounds, such as providing a moist wound-healing environment, absorbing wound exudates, and providing appropriate oxygen permeability. Natural polymers (such as chitosan and sodium alginate) and synthetic polymers (such as polypeptide, polyethylene glycol, polyacrylic acid, and polyvinyl alcohol) are widely used as building blocks for constructing polymer hydrogels [9,10]. These polymer-based hydrogels often use toxic cross-linking agents or heavy metal ions and require complex synthesis steps and delicately designed monomers, which greatly limits the large-scale production and biological application [11].

Different from polymer gel (usually formed by cross-linking of polymer chains through a covalent bond), supramolecular gel is formed by noncovalent bond forces between low-molecular weight gelators (LMWGs) such as hydrogen bonds, π–π stacking forces, the van der Waals force, and hydrophobic forces [12,13]. Due to the programmable structure–property relationship, supramolecular gel as a functional material is widely used in antibacterial, anticancer, three-dimensional cell culture, cell proliferation, and tissue-engineering fields, which has aroused great research enthusiasm among researchers [14,15,16,17]. Among the numerous materials used to construct supramolecular gel, polypeptides and oligopeptides, as well as amino acids and their derivatives, have received great attention and are widely used to construct supramolecular hydrogels for biomedical applications due to these substances’ excellent biocompatibility, biodegradability, and programmable design, which endows versatility [18,19]. Most supramolecular antibacterial gels are constructed by loading antibiotics [20,21]. Bajaj et al. constructed a ciprofloxacin hydrochloride (CIP·HCl)-loaded hydrogel by nonimmunogenic cholic acid–glycine–glycine conjugate, which exhibited excellent antimicrobial effects [22]. Kumar et al. developed CIP·HCl-loaded supramolecular hydrogels based on glyoxylamide, which showed good antibacterial activity by a sustained release of CIP·HCl [23]. Unfortunately, the antibiotic-loaded gel is prone to multidrug resistance due to the inability to rapidly release and maintain dose concentration of antibiotics at the site of bacterial infection.

A stimulus-responsive drug-delivery system could release drugs rapidly at targeted sites, maintain effective dose concentration, improve the treatment effect, and prevent the emergence of multidrug resistance. Bacterial-infected wounds have a microenvironment of low pH and a high concentration of ROS [24], which can be used as an endogenous stimulus for stimulus-responsive drug-delivery systems. Recently, developing ROS-responsive supramolecular gel for antitumor, antibacterial, and wound dressing has aroused great attention [25,26,27]. Yang et al. developed a *L*-arginine and H_2_O_2_ co-loaded hydrogel based on polyvinyl alcohol and N^1^-(4-Boronobenzyl)-N^3^-(4-Boronophenyl)-N^1^,N^1^,N^3^,N^3^-Tetramethyl-1,3-Propanediaminium, which could allow for the ROS-responsive release of nitric oxide to kill bacterial and promote wound healing [28]. To the best of our knowledge, there is no report on the construction of ROS-responsive antibacterial supramolecular gel based on LMWGs.

Furthermore, supramolecular hydrogel with inherent antibacterial properties is a good alternative to antibiotics, which can effectively reduce the use of antibiotics and kill antibiotic-resistant bacteria [29]. Phenylalanine is widely used in the construction of supramolecular gel due to its good biocompatibility and excellent building block of gelator (it has strong hydrogen bonding and π–π forces sites.) [30]. Excitingly, supramolecular gel based on phenylalanine has inherent antibacterial properties [31,32]. Thakur et al. reported that both Fmoc–phenylalanine hydrogel and the Fmoc–phenylalanine solution have antibacterial activity against gram-positive bacterial [33]. Marchesan et al. developed a supramolecular hydrogel based on the self-assembly of *N*-(4-Nitrobenzoyl)-Phenylalanine, which exhibited a mild antimicrobial activity against *E. coli* [34]. Hydrazide and its derivatives have received more attention in the field of drug research and development in recent years due to their biological activities (antibacterial and anti-inflammatory) [35,36,37]. Due to hydrazide’s strong hydrophilicity and multiple hydrogen-bonding sites, hydrazide groups are widely used as building blocks to regulate the hydrophilicity and hydrophobicity balance of compounds to construct supramolecular hydrogels [38,39,40]. Smith et al. developed a supramolecular hydrogel based on the hydrazide compound, which can react with aldehydes without disrupting the gel network and selectively reacted with specific aldehydes in aldehyde mixtures [41]. However, inherent antibacterial properties of supramolecular gel based on hydrazide compounds have been ignored. Hence, the design of LMWGs integrating hydrazide and phenylalanine is an effective method to construct supramolecular gel with inherent antibacterial properties.

Herein, we designed a kind of ROS-responsive LMWG, which could self-assemble into supramolecular gel with an inherent antibacterial performance. The ROS-responsive LMWGs were constructed by conjugating phenylalanine methyl ester to both ends of thioketal with different alkyl chain lengths before they react with hydrazine hydrate. Gelation properties and critical gel concentration (CGC) of these gelators were investigated in multiple solvents. The self-assembly mechanism of gelator was investigated by ^1^H NMR and FT–IR spectroscopy. Rheological properties of the supramolecular gel were studied by rheometer. ^1^H NMR and the in vitro release experiment were chosen to evaluate whether the supramolecular gel has ROS-responsive degradation performance. The biological safety of the supramolecular gel was evaluated by H929 and the 293T cell. The antibacterial property of supramolecular gel against *S. aureus* and *E. coli* was investigated by the agar disk experiment and the bacterial adhesion experiment.

## 2. Experimental Section

### 2.1. Materials

All solvents and reagents used in this study were chemically pure. *N,N’*-carbonyldiimidazole (CDI), *L*-Phenylalanine methyl ester hydrochloride, 6-bromocaproic acid, and 11-bromoundecanoic acid were provided from Saen chemical technology (Shanghai, China) Co., Ltd. Cell counting kit-8 (CCK-8), 3-mercaptopropionic acid, and methylthiazoletetrazolium (MTT) were purchased from Aladdin Bio–Chem Technology (Shanghai, China) Co., Ltd. Poly(ethylene glycol) (*M*_w_ = 200 g/mol or 400 g/mol) was bought from Sigma-Aldrich Co., Ltd. Solvents (St. Louis, MO, USA), CIP·HCl, levofloxacin hydrochloride (LFX·HCl), and tetracycline hydrochloride (TCC·HCl) were bought from Shanghai Titan Technology Co., Ltd. (Shanghai, China). Hydrogen peroxide (H_2_O_2_) and hydrazine hydrate were bought from Sinopharm Chemical Reagent Co., Ltd. (Shanghai, China).

### 2.2. Characterizations

Chemical structure of the synthesized compounds was measured by ^1^H NMR spectroscopy (Bruker Avance II NMR spectrometer, Billerica, MA, USA, 500 MHz). Molecule weight of new compounds was determined by HRMS (Thermo Scientific Q Exactive LCMS, Waltham, MA, USA). Scanning electron microscope (SEM, Magellan 3020, Thermo, American) was employed to observe the microstructure of xerogel. Gelation mechanism was studied by FT–IR (Nicolet 6700, American) and ^1^H NMR spectra. Microplate reader (Multiskan GO Microplate Spectrophotometer, Thermo Scientific) was applied to measure drug-releasing amount and absorbance of CCK-8 and MTT. Mechanical properties of gel were measured by a rheometer (DiscoveryHR20, American).

### 2.3. Synthesis of 6-Mercaptocaproic Acid/11-Mercaptoundecanoic Acid

According to reported method, 6-bromocaproic acid and 11-mercaptoundecanoic acid were prepared [42].

### 2.4. Synthesis of Thioketal with Different Length Alkyl Chains

Thioketal TK1 (n = 2), TK2 (n = 5), and TK3 (n = 10) with different lengths of alkyl chains were synthesized according to the procedure reported in our previously work [43].

### 2.5. Synthesis Conjugate of Phenylalanine and Thioketal

Triethylamine (3.70 mL, 26.4 mmol) was added to the dichloromethane solution of *L*-phenylalanine methyl ester hydrochloric acid (4.30 g, 22 mmol), and the hydrochloric acid molecules were removed by acid-base reaction. TK1 (2.52 g, 10 mmol) and CDI (3.57 g, 22 mmol) were dissolved in dichloromethane (25 mL) and stirred until no more bubbles are generated. Subsequently, the activated thioketal solution was added to *L*-Phenylalanine methyl ester solution and stirred for 24 h under protection of N_2_. After the solvent was removed, tetrahydrofuran was added to dissolve the product, and the undissolved triethylamine hydrochloride was removed by filtering. The filtrate was concentrated and purified by column chromatography to obtain the product Phe–TK1–Phe (4.82 g; yield: 84.1%). The other conjugate of phenylalanine and TK2/TK3 was synthesized similarly.

### 2.6. Synthesis of ROS-Responsive Gelator

Phe–TK1–Phe (2.87 g, 5 mmol) was dissolved in mixture solvent (30 mL) of dichloromethane and methanol (*v*:*v* = 1:2). Then, the solution was protected by N_2_. Subsequently, hydrazine hydrate (2 mL) was injected by a syringe, and the mixed solution was stirred for 24 h under ice bath conditions. The mixture was filtered and the solid was washed by dichloromethane several times. The solid was collected and vacuum-dried to obtain the gelator 1 (2.58 g, yield, 90.1%). Gelator 2 (n = 5) and Gelator 3 (n = 10) were synthesized similarly to the procedure of Gelator 1.

### 2.7. ROS-Responsiveness of the Gelators

ROS-responsiveness of gelator was measured by ^1^H NMR spectra. Then, 200 mM of H_2_O_2_ solution was obtained by adding 0.2 mL of H_2_O_2_ (30%) to 9.8 mL of PBS (pH 7.4). Gelator 3 (20 mg) was dissolved in 5 mL of acetone then mixed with H_2_O_2_ (200 mM, 10 mL) by vortex. After it was incubated at 37 °C for 24 h, the mixed solution was lyophilized, and the lyophilized powder was characterized by ^1^H NMR.

### 2.8. Preparation of Blank and Antibiotic-Loaded Supramolecular Gel 

First, 1 mL of solvent (such as H_2_O or chloroform) and certain amount of gelator were added to a screw bottle then heated until the gelator is completely dissolved. The formation of gel was judged by inverted observation, which determined the minimum amount of gelator required to form a complete gel; this value was critical gel concentration (CGC).

Drug-loaded supramolecular gel was obtained by similar procedures of prepared blank gel. Gelator and antibiotic (CIP·HCl, LFX·HCl, TCC·HCl) were added to a mixture solvent of H_2_O and PEG200 (1 mL, *v*:*v* = 2:3), then heated until the solution becomes clear. The antibiotic-loaded gel was formed as the temperature of solution cooling to RT. Supramolecular gel constructed by 20 mg of Gelator 3 in 1 mL of mixture solvent (PEG200 and H_2_O) was chosen to assess the antibiotic-loading properties. The maximum antibiotic-loading content is the limit value when slightly more antibiotic cannot form gel. Antibiotic-loading content (ALC) was obtained through the following formula:

ALC = [weight of antibiotic/(weight of antibiotic and gelator)] × 100%

### 2.9. Characterization of Microstructure of Gel

Microstructure of gel was observed by SEM. A certain amount of gelator was added to chloroform or ethanol to prepare gel. The gel was dried by oil pump at RT to prepare xerogel. The microstructure of the xerogel was observed by SEM after gold sputtering. 

### 2.10. Investigation of Gelation Mechanism of Gelator

FT–IR and ^1^H NMR were used to study the gelation mechanism of gelator. For FT–IR: Preparation of chloroform gel by Gelator 3 with a concentration of 10, 25 mg/mL, respectively. The chloroform gel was dried by reducing pressure through oil pump to obtain xerogel. Subsequently, the xerogel/gelator was measured by FT–IR. For ^1^H NMR: A particular amount of Gelator 3 was added to deuterated chloroform to prepare gel with gelator concentration of 10, 20, 25 mg/mL, respectively. These gels were characterized by ^1^H NMR.

### 2.11. Rheological Measurement

Rheological properties of gel were characterized by rheometer. The specific experimental method is similar to our previous work [25].

### 2.12. In Vitro Drug Release

Neutral PBS (pH 7.4) with or without ROS reagent (H_2_O_2_) was chosen to simulate physiological environment and microenvironment of bacterial-infected wounds to study the in vitro drug-release properties of CIP·HCl-loaded supramolecular gel. Gelator 3 (20 mg) and CIP·HCl (1 mg) were added to threaded bottles containing mixed solvent of PEG200 and water (1 mL, *v*/*v* = 2:3) to prepare CIP·HCl-loaded supramolecular gel. Then, 2 mL of release medium were carefully added to the top of gel before the screw bottle was placed in a constant temperature water bath oscillator (37 °C) for 72 h. Next, 0.2 mL of release solution was obtained at the predetermined time points and added to 0.2 mL of fresh medium simultaneously. Concentration of CIP·HCl in the release solution was measured by a microplate reader at the wavelength of 273 nm, and the cumulative release amount of CIP·HCl in the release solution at each time point was calculated. Three parallel experiments were performed for each sample.

### 2.13. Cytotoxicity Assay

Cytotoxicity of supramolecular gel was tested by a CCK-8 assay using L929 and 293T cells [18]. Prepare supramolecular gels by Gelator 2 with a concentration of 25 mg/mL in mixture solvent of PEG200 and H_2_O (*v*:*v* = 1:1), as well as Gelator 3 with a concentration of 20 mg/mL in mixture solvent of PEG200 and H_2_O (*v*:*v* = 3:2). In addition, 100 μL of sol were added into 96 well plates and set overnight to form gel. Cells were seeded atop the supramolecular gels and incubated for 24 h. After removal of the cell medium, 100 μL of fresh medium containing 10 μL CCK-8 solutions were added into each well and then incubated at 37 °C for 2 h. Control wells included cell-free gels, cells, and PBS. Optical density (OD) values at 450 nm were recorded using a microplate reader. Viability of cells was calculated according to the following equation:

Viability% = (Aa − Ab)/(Ac − Ad) × 100%

Aa: OD values of mixtures of hydrogel, CCK-8 and cells at 450 nm; Ab: OD values of mixtures of hydrogel and CCK-8 mixture at 450 nm; Ac: OD values of mixtures of cells and CCK-8 at 450 nm. Ad: OD values of CCK-8 at 450 nm.

### 2.14. Bacterial Adhesion Assay

Antibacterial capacity of the supramolecular gels was evaluated by *E. coli* (ATCC (Manassas, VA, USA) 25922) and *S. aureus* (ATCC 43300), which cultured with tryptic soy broth and agar. Then, 100 μL of sol were added to 96 well plates in triplicate and set overnight to form gel. Subsequently, 100 μL of *E. coli* or *S. aureus* at a bacteria density of 1 × 10^7^ CFU/mL were incubated with gel for 24 h at 37 °C. The antibacterial activity of gels was assessed according to our previous work [44].

### 2.15. The Spread Plate Tests

*E. coli* and *S. aureus* bacteria cells (1 × 10^7^ CFU/mL) were incubated on supra-molecular gels for 24 h and washed three times with PBS. Then, 100 μL of diluted bacteria solution (1000 times) were incubated on agar plates for 24 h. Bacterial colonies were photographed by Colony Counting Instrument, and the antibacterial ratio was counted by following formula:

$$Antibacterial\;ratio=\frac{{CFU}_{control\;group}-{CFU}_{experimental\;group}}{{CFU}_{control\;group}}\times 100\%$$

## 3. Results and Discussion

### 3.1. Synthesis and Characterization of Gelator 

**Scheme 1:**
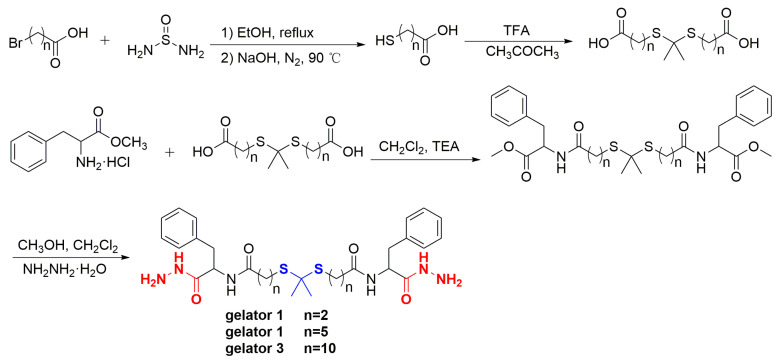
Synthesis scheme of gelators.

ROS-responsive LMWGs were synthesized according to Scheme 1. The structure of intermediates and gelators was characterized by ^1^H NMR. A new single peak (-C*(CH*_3_*)*_2_-) appeared at 1.604 ppm (Figure S1), 1.577 ppm (Figure S2), and 1.582 ppm (Figure S3), respectively, and the other peaks could be well-matched with the structure of target thioketal, which proved that the thioketal was successfully prepared. The dd peak appeared at 4.910 ppm (Figure S4), 4.895 ppm (Figure S5), and 4.903 ppm (Figure S6), respectively, which was the peak of typical methine proton (PhCH_2_*CH*-), which proved that *L*-phenylalanine methyl ester has successfully conjugated to thioketal. The single peak of methoxyl protons (*CH*_3_O-) appeared at 3.718 ppm (Figure S4), 3.723 ppm (Figure S5), and 3.730 ppm (Figure S6), respectively, while disappeared in the spectrum of corresponding gelators. Furthermore, the other peaks could be well-assigned to the structure of the target gelator, which demonstrated that Gelator 1, Gelator 2, and Gelator 3 were successfully obtained.

HRMS was used to further characterize the structure of new compounds. **Compound TK2**, HRMS (ESI^+^) calcd for (C_15_H_28_O_4_ S_2_ + Na)^+^: 359.13212; found: 359.13129. **Compound TK3**, HRMS (ESI^+^) calcd for (C_25_H_48_O_4_S_2_ + Na)^+^: 499.28862; found: 499.28787. **Compound Phe-TK1-Phe**, HRMS (ESI^+^) calcd for (C_29_H_38_N_2_O_6_S_2_ + H)^+^: 575.22440; found: 575.22382. **Compound Phe-TK2-Phe**, HRMS (ESI^+^) calcd for (C_35_H_50_N_2_O_6_S_2_ + H)^+^: 659.31831; found: 659.31750. **Compound Phe-TK3-Phe**, HRMS (ESI^+^) calcd for (C_45_H_70_N_2_O_6_S_2_ + H)^+^: 799.47481; found: 799.47382. **gelator 1**, HRMS (ESI^+^) calcd for (C_27_H_38_N_6_O_4_ + H)^+^: 575.24687; found: 575.24628. **gelator 2**, HRMS (ESI^+^) calcd for (C_33_H_50_N_6_O_4_ + H)^+^: 659.34077; found: 659.34003. **gelator 3**, HRMS (ESI^+^) calcd for (C_43_H_70_N_6_O_4_ + H)^+^: 799.49727; found: 799.49762.

### 3.2. Preparation of Blank Supramolecular Gel and Antibiotic-Loaded Supramolecular Gel

**Figure 1:**
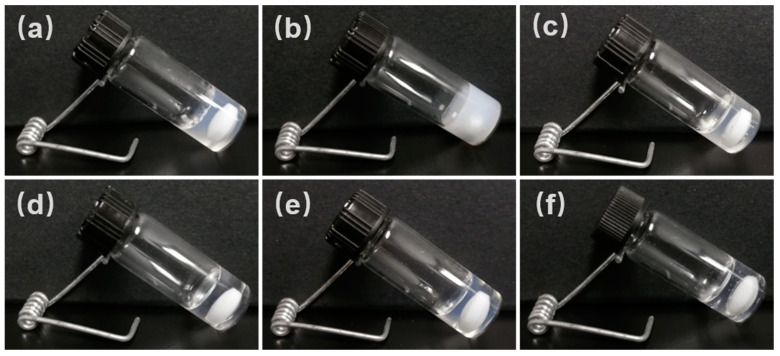
Photos of different gels. (**a**) Ethanol gel prepared by Gelator 3 (15 mg/mL) in ethanol; (**b**) Gel constructed by Gelator 3 (15 mg/mL) in mixture solvents of ethanol and H_2_O (*v*:*v* = 1:1); (**c**) Gel prepared by Gelator 3 (15 mg/mL) in PEG200; (**d**) Chloroform gel prepared by Gelator 3 (10 mg/mL) in chloroform; (**e**) Acetonitrile gel prepared by Gelator 3 (2 mg/mL) in acetonitrile; (**f**) Ethylene glycol gel prepared by Gelator 3 (5 mg/mL) in ethylene glycol.

The gelation properties of Gelator 1, Gelator 2, and Gelator 3 were widely explored in 18 solvents from weak polarity solvents (petroleum ether, cyclohexane, etc.) to strong polarity (acetonitrile, acetone, methanol, water, etc.) solvents and mixed solvents (PEG200 and water, ethanol and water). Some photos of gel are shown in Figure 1. The CGC of Gelator 1, Gelator 2, and Gelator 3 in different solvents was determined (Table S1). All gelators can form gel in ethyl acetate, dichloromethane, chloroform, acetonitrile, methanol, and a mixture of ethanol and H_2_O, which indicated that all gelators have good gelation ability. Compared to Gelator 1 and Gelator 2, Gelator 3 could form gel in more solvents. In addition, Gelator 3 has the lowest CGC among three gelators in the same solvent. These results indicated Gelator 3 has the best gelation ability. A good gelling performance means that a more stable gel can be obtained, and that the gel has a higher antibiotic-loading capacity. Therefore, the supramolecular gel, which was constructed by Gelator 3, was chosen to investigate the antibiotic-loading capacity, gelation mechanism, rheological properties, ROS-responsive properties, in vitro drug-release properties of antibiotics-loaded gel, and antibacterial activity. 

The combined administration of multiple drugs targeting different sites is an effective way to improve treatment efficacy and avoid drug resistance. The construction of the antibiotic-loading supramolecular gel by supramolecular gel with inherent antibacterial properties can not only prevent the development of bacteria resistance but also effectively kill drug-resistant bacteria. CIP·HCl, LFX·HCl, and TCC·HCl were chosen to evaluate the antibiotic-loading capacity of supramolecular gel. Since both PEG and water are biocompatible, and since the gelator cannot form gel in water, the antibiotics have poor solubility in PEG200. Hence, the mixture solvent of PEG200 and H_2_O was chosen to prepare an antibiotic-loaded gel. Supramolecular gel exhibited an excellent antibiotic-loading capacity; the ALC of CIP·HCl, LFX·HCl, and TCC·HCl were 84.6%, 87.5%, and 63.6%, respectively.

### 3.3. Characterization of Microstructure of Supramolecular Gel

**Figure 2:**
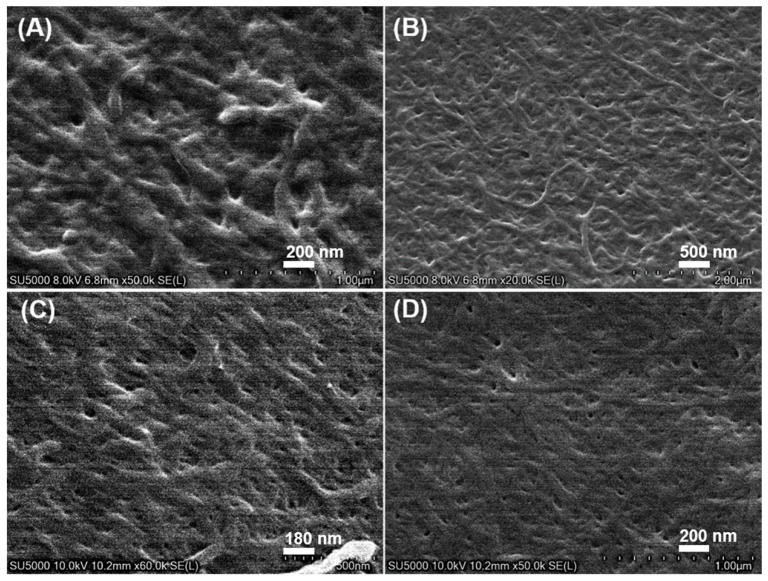
SEM images of xerogel. (**A**,**B**) were chloroform gel of Gelator 3 with a concentration of 10 mg/mL and 15 mg/mL, respectively; (**C**,**D**) were ethanol gel of Gelator 3 with a concentration of 10 mg/mL and 15 mg/mL, respectively.

SEM was applied to characterize the microstructure of the supramolecular gel. Since SEM is not suitable for the direct observation of wet samples, the xerogel was prepared to reduce the pressure through the oil pump. The gel prepared in a low-boiling-point solvent (such as ethanol or chloroform) was chosen for SEM observation. A dense and entangled fiber network was observed in the xerogel of ethanol and chloroform with different concentrations (Figure 2), which indicated that Gelator 3 will first self-assemble into one-dimensional fiber before the fibers interweave and intertwine to form a cross-linked three-dimensional network; the solvent was then fixed in it. The results suggested Gelator 3 could self-assemble into supramolecular gel by non-covalent bond interactions in ethanol and chloroform.

### 3.4. Investigation of ROS-Responsive Degradation Properties

**Figure 3:**
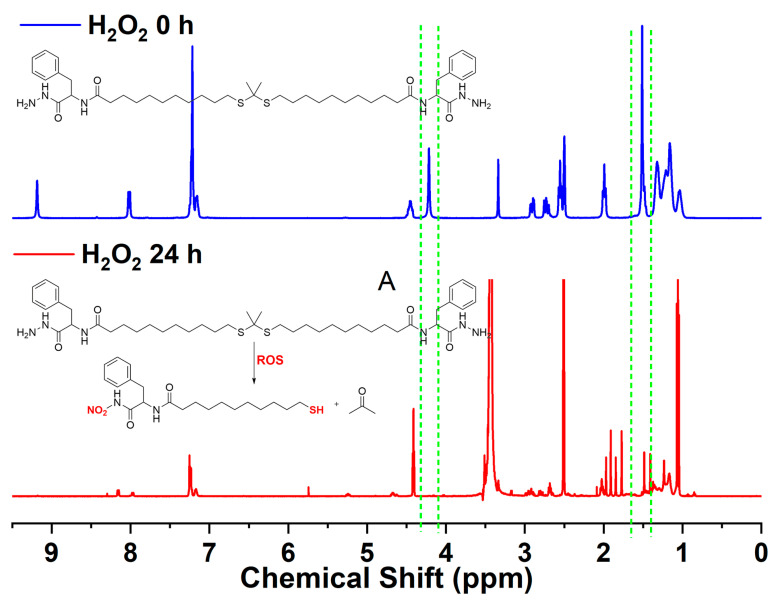
^1^H NMR spectra of Gelator 3 before and after treated with H_2_O_2_ solution.

^1^H NMR was applied to characterize the ROS-responsive properties of supramolecular gel. The H_2_O_2_ solution (200 mM) was used as an ROS reagent. The peak of methyl protons (1.51 ppm) and amino protons (4.22 ppm) disappeared after they were treated with H_2_O_2_ solution for 24 h (Figure 3), which indicated that the thioketal structure of Gelator 3 has been cleaved and the amino has been oxidized [43]. The results proved that Gelator 3 has ROS-responsive properties.

### 3.5. Self-Assembly Mechanism of Gelator

**Figure 4:**
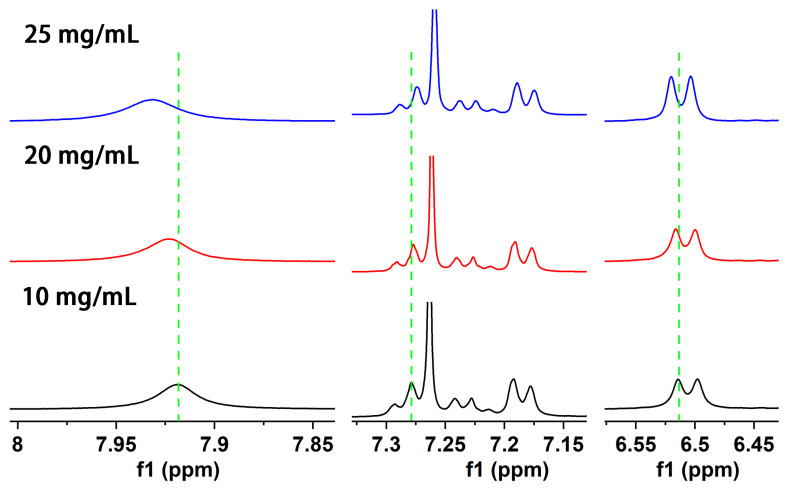
^1^H NMR spectra of Gelator 3 with different concentrations in chloroform-d.

In order to explore the self-assembly mechanism of gelator, different concentrations of chloroform-d gel were prepared and measured by ^1^H NMR. As the concentration of Gelator 3 increases from 10 mg/mL to 25 mg/mL, the chemical shift of the amide N–H bond proton significantly moves to the low field (from 7.919 ppm shift to 7.931, 6.514 ppm to 6.520 ppm, Figure 4). It demonstrated that the hydrogen-bonding force is present among gelators. With the increase of gelator concentration, more gelator molecules participated in the formation of hydrogen bonds, and the degree of hydrogen-bond association is higher [45,46]. Moreover, the chemical shift of benzene ring protons moved to the high field (from 7.279 ppm to 7.277 ppm) with the increase of gelator concentration, which indicated that the π–π stacking force is present among gelators [29]. In conclusion, the hydrogen bond and π–π stacking force are the self-assembly driving force of gelator.

**Figure 5:**
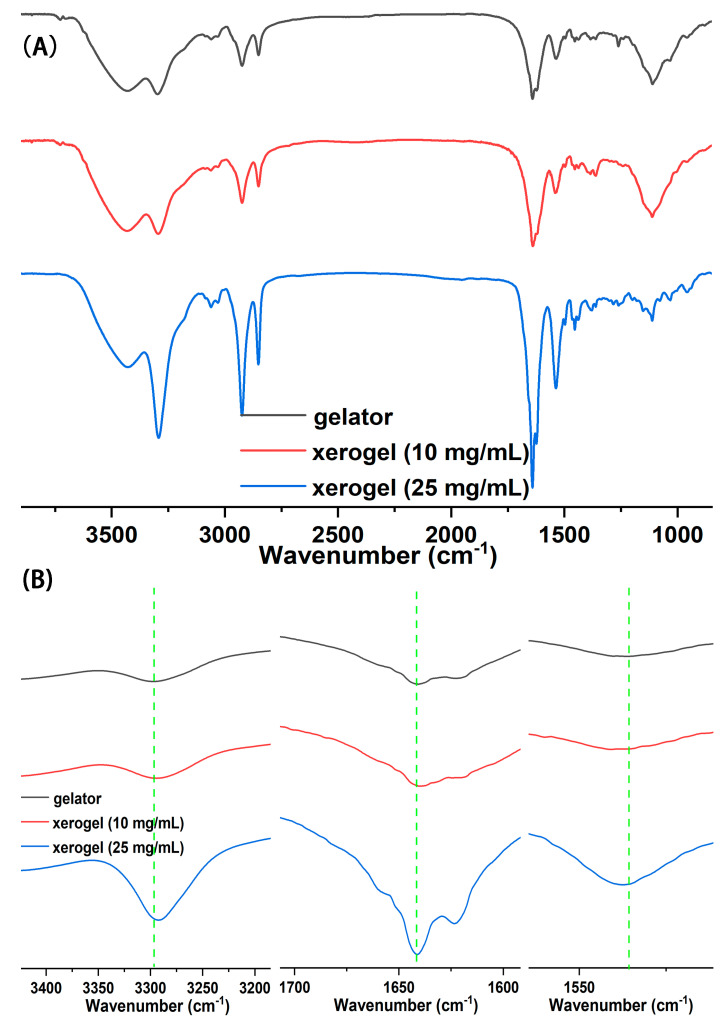
(**A**) FT–IR of Gelator 3 and xerogel; (**B**) Enlarged FT–IR of Gelator 3 and xerogel; Xerogel was obtained by drying the chloroform gel with different concentrations.

FT–IR spectroscopy was applied to further disclose the self-assembly driving force of gelator. In the spectrum of Gelator 3 (Figure 5A), the peaks at 3433.7 cm^−1^ and 3296.5 cm^−1^ separately were asymmetrical and symmetrical stretching vibrations of the N–H bond in the amnio group; the peak at 2926.7 cm^−1^ and 2846.6 cm^−1^ separately were asymmetrical and symmetrical stretching vibrations of methylene; the peak at 1641.6 cm^−1^ and 1534.8 cm^−1^ belonged to stretching vibrations of carbonyl and the deformation vibrations of the N–H bond, respectively. The stretching vibration peak of the N–H bond in the amino group in the spectrum of xerogel shifted to a low wavenumber compared with the spectrum of Gelator 3, and from 3296.5 cm^−1^ (Gelator 3), it shifted to 3293.8 cm^−1^ (10 mg/mL) and 3291.1 cm^−1^ (25 mg/mL) (Figure 5B). The results demonstrated that a strong hydrogen-bond force exists in the gel phase [46]. Compared to the spectrum of the gelator, the frequency of the carbonyl-stretching vibration peak of xerogel also slightly shifted from a low wavenumber (from 1641.6 cm^−1^ (gelator) to 1640.8 cm^−1^ (25 mg/mL)), while the frequency of the N–H deformation vibration peak of xerogel appeared at a high wavenumber (1536.6 cm^−1^ (10 mg/mL), 1537.2 cm^−1^ (25 mg/mL)). The results indicated that the hydrogen bond formed between carbonyl and the N–H bond [38]. The spectral band of the methylene-stretching vibration peak in xerogel was stronger than in gelator, which indicated that the alkyl chains between gelators in the gel phase are stacked, and that Van der Waals forces exist between alkyl chains. In summary, hydrogen and the van der Waals force are important driving forces for gelator self-assembly.

### 3.6. Rheological Properties

**Figure 6:**
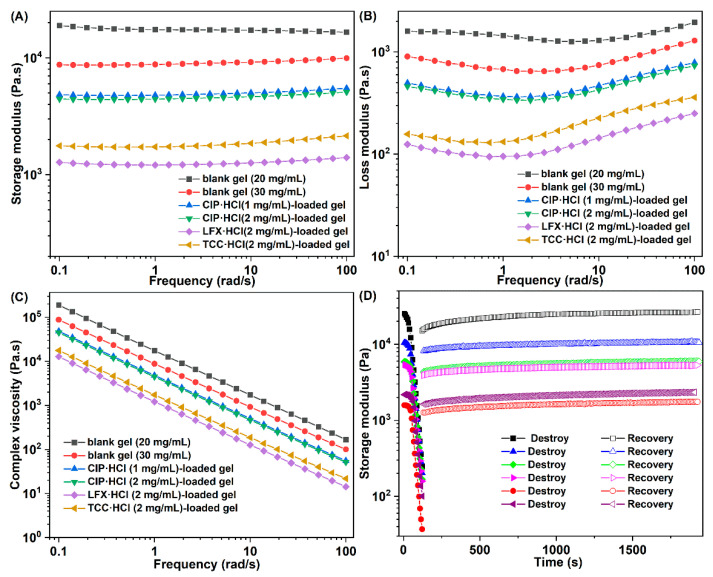
Rheological properties of supramolecular gel: storage modulus (**A**), loss modulus (**B**), and dynamic complex viscosity (**C**) as a function of angular frequency of the gels; (**D**) Destruction and recovery of the gels.

Mechanical properties of both blank gels and antibiotic-loaded gels were investigated by rheometer [22]. The storage modulus of gels higher than the corresponding loss modulus was observed in Figure 6A,B, which confirmed that all gel samples that were applied for the rheological test were real gels. The dynamic modulus of the blank gel increased as the concentration of Gelator 3 increased from 20 to 30 mg/mL, which implied that the mechanical strength of the gels increased as the concentration increased [47]. It may be caused by more gelator molecules participating in self-assembly and forming stronger gel with the increased concentration of gelator. The dnamic modulus of CIP·HCl-loaded gel, LFX·HCl-loaded gel, and TCC·HCl-loaded gel were obviously decreased compared to their corresponding blank gels, which indicated that the antibiotic molecule will disturb the self-assembly of gelator (Figure 6A). Moreover, with the increase of the CIP·HCl-loading capacity, the dynamic modulus of the CIP·HCl-loaded gel slightly decreased (Figure 6A). Complex viscosities of all gels decreased linearly with increasing frequency (Figure 6C), which indicated that both blank and antibiotic-loaded gels have shear-thinning performances [25]. Results of the dynamic-strain and time-sweep experiment (Figure 6D) showed that the network of gel was destroyed as the strain increased; however, it could be recovered after the strain was withdrawn. The destroy-and-recovery properties of these gels was attributed to the intrinsic dynamic and reversible non-covalent interaction between gelators [25]. The excellent shear-thinning and destroy-and-recovery ability ensure that the blank and antibiotic loaded-gels have injectability.

### 3.7. In Vitro Drug-Release Properties

**Figure 7:**
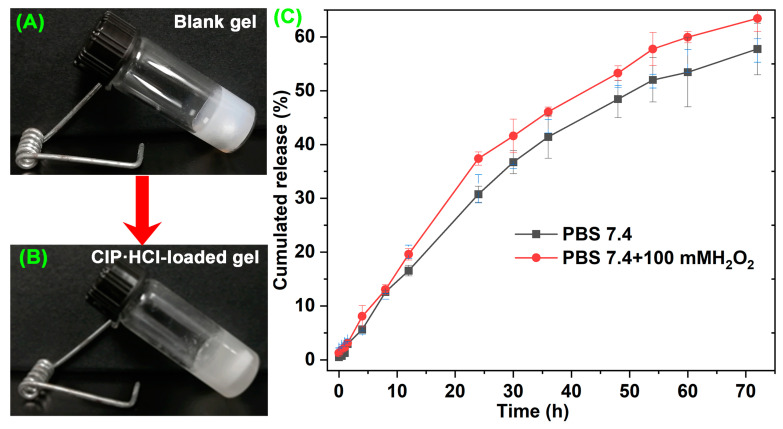
Images of blank gel (**A**) and CIP·HCl-loaded gel (**B**); (**C**) Drug-release profiles of CIP·HCl-loaded Gelator 3 gel in the environment of PBS 7.4 and PBS7.4 with 100 mM of H_2_O_2_.

Drug-release properties are a crucial parameter for evaluating carrier performance. Neutral PBS and neutral PBS with 100 mM H_2_O_2_ were chosen to evaluate the in vitro drug-release properties of antibiotic-loaded supramolecular gel. There were no significant differences in the release curves between the two environments during the first 10 h of release (Figure 7C). Subsequently, the CIP·HCl-loaded supramolecular gel in the ROS environment exhibited a faster drug-release rate. In the initial stage, the antibiotics were likely loaded on the gel surface and released rapidly, which is a passive diffusion process that results in similar release curves in the two media. Then, the antibiotics encapsulated in gel began to release. In this stage, the gel in the ROS medium could obtain an accelerated release through the ROS-responsive degradation. The cumulated release amount of CIP·HCl-loaded supramolecular gel in the neutral and ROS environment were 57.75% and 63.44%, respectively (Figure 7C). The results indicated that the supramolecular gel has ROS-responsive in vitro drug-release properties.

### 3.8. Cytotoxicity Test

**Figure 8:**
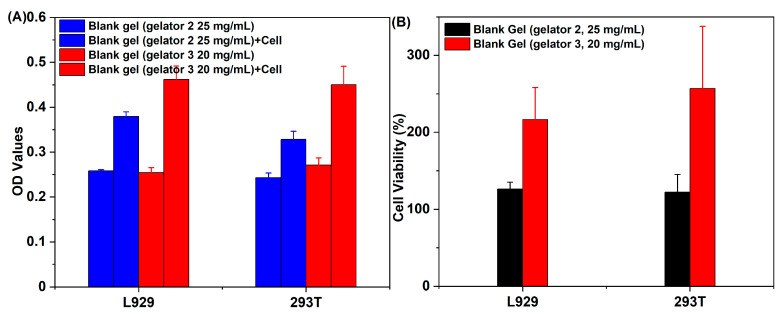
(**A**) OD values of blank gel and blank gel cultured with L929 and 293T cells; (**B**) Cytotoxicity of ROS-responsive blank gel incubated with L929 and 293T cells.

Good biocompatibility is a prerequisite for supramolecular gel to be used as drug-delivery vehicles. Since Gelator 1 cannot form gel in biocompatibility solvents (such as PEG200, H_2_O, and the mixture solvent of PEG200 and H_2_O), the cytotoxicity of supramolecular gel constructed by Gelator 2 and Gelator 3 in the mixture solvent of PEG200 and H_2_O was investigated against L929 and 293T cells. The group of gel and cell co-culture showed higher OD values than the group of gel, and the supramolecular gel constructed by Gelator 3 exhibited higher OD values than supramolecular gel constructed by Gelator 3 (Figure 8A). In addition, the cell viabilities treated with supramolecular gels were higher than 100% at L929 and 293T cells, especially the supramolecular gels constructed by Gelator 3 (Figure 8B). The results suggested that the supramolecular gels not only have good biocompatibility but also show that they promote cell proliferation.

### 3.9. In Vitro Antibacterial Test

**Figure 9:**
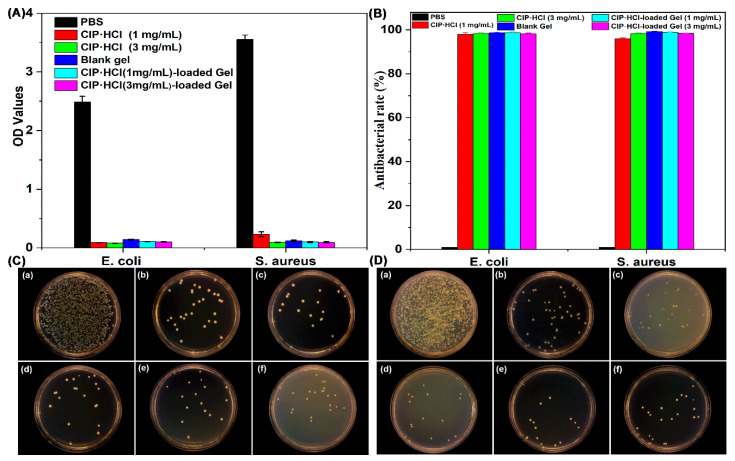
(**A**) Bacterial viability of *E. coli* and *S. aureus* cultured on different materials (PBS, CIP·HCl, blank gel, CIP·HCl-loaded gel); (**B**) Antibacterial rate of the samples calculated from (**C**,**D**); *E. coli colonies* (**C**) and *S. aureus colonies* (**D**) treated with PBS (a), CIP·HCl (b, 1 mg/mL), CIP·HCl (c, 3 mg/mL), blank gel (d), CIP·HCl-loaded gel (e, 1 mg/mL), and CIP·HCl-loaded gel (f, 1 mg/mL).

*E. coli* and *S. aureus* bacterial cells were selected to evaluate the in vitro antibacterial activity of supramolecular gel. Since Gelator 3 has better comprehensive performance than Gelator 2, Gelator 3 with a concentration (20 mg/mL) slightly higher than CGC (18 mg/mL) was chosen for antibacterial investigation. Antibacterial properties of supramolecular gel were first evaluated by bacterial adhesion assay. Bacterial activity was assessed by MTT assay after incubation with gel for 24 h. The viability of both *E. coli* and *S. aureus* treated with CIP·HCl, blank gel, and CIP·HCl-loaded gel was comparable, while it was significantly lower when treated with PBS (Figure 9A), which indicated both blank gel and CIP·HCl-loaded gel have excellent antibacterial properties.

Antibacterial properties of the supramolecular gel were further investigated by the colony formation unit (CFU) assay. The numbers of *E. coli* and *S. aureu* colonies formed on the agar plates of different supramolecular gels were shown in Figure 9C,D. An obvious decrease of the number of colonies was observed for antibiotic (CIP·HCl), blank supramolecular gel, and CIP·HCl-loaded supramolecular gel compared with control group (PBS), thus emphasizing higher bactericidal activity of antibiotic (CIP·HCl), blank supramolecular gel, and CIP·HCl-loaded supramolecular gel. The antibacterial rate of CIP·HCl (1 mg/mL), CIP·HCl (3 mg/mL), blank supramolecular gel, CIP·HCl (1 mg/mL)-loaded supramolecular gel, and CIP·HCl (3 mg/mL)-loaded supramolecular gel, separately, were 97.96%, 98.30%, 98.65%, 98.75%, and 98.21%, respectively, against E. coli, while they were 95.90%, 98.30%, 99.12%, 98.86%, and 98.36%, respectively, against S. aureus (Figure 9B). The antibacterial performance of blank supramolecular gel was equivalent to CIP·HCl and CIP·HCl-loaded supramolecular gel. The results indicated that the supramolecular gel constructed by Gelator 3 has inherent potent antibacterial properties. The antibacterial properties of blank supramolecular gel come from Gelator 3, as PEG200 and H_2_O are biocompatible and have no antibacterial properties. The innate and excellent antibacterial properties of Gelator 3 probably originate from the moieties of phenylalanine and hydrazide in its structure, which possess innate antibacterial properties. It may be attributed to the combined antibacterial effects of phenylalanine and hydrazide, resulting in comparable antibacterial effects to antibiotics for the supramolecular gel. Due to the good antibacterial properties of both the blank gel and antibiotics themselves, the antibiotic-loaded gel also exhibits excellent antibacterial effects. The supramolecular gel as a novel antibacterial agent has great application prospects in treating wounds infected with antibiotic-resistant bacterial. The antibiotic-loaded ROS-responsive supramolecular gel, as a novel antibacterial agent, has great potential in antibacterial and preventing the development of bacterial resistance because it can efficiently and cooperatively kill bacterial through the gelator itself and the ROS-responsive release of antibiotics.

## 4. Conclusions

A kind of ROS-responsive LMWG was synthesized, while the gelation properties and CGC were explored. Gelator 3 with the best gelation properties was chosen to investigate the antibiotic-loading capacity, gelation mechanism, rheological properties, ROS-responsive properties, in vitro drug-release properties of antibiotic-loaded gel, cytotoxicity, and antibacterial activity. Results showed that Gelator 3 has ROS-responsive degradation properties and a good antibiotic-loading capacity. Results of ^1^H NMR and FT–IR experiments revealed that the hydrogen bond, π–π stacking force, and van der Waals force are self-assembling driving forces of gelator. The blank gel and CIP·HCl-loaded gel exhibited good shear-thinning properties. The cytotoxicity assay indicated supramolecular gel constructed by Gelator 3 in PEG200 and H_2_O have good biocompatibility and promote cell-proliferation properties. Results of in vitro antibacterial experiments showed that the supramolecular gel has innate antibacterial properties, and the antibacterial properties of the supramolecular gel were equivalent to antibiotic CIP·HCl and CIP·HCl-loaded gel. Our supramolecular gel and antibiotic-loaded supramolecular gel as a wound-dressing have enormous potential in treating antibiotic-resistant bacteria-infected wounds.

## Data Availability

The data that support the findings of this study are available in the supplementary material of this article.

## Supplementary Materials

The following supporting information can be downloaded at: https://www.mdpi.com/article/10.3390/pharmaceutics15082161/s1, Figure S1: ^1^H NMR spectra of TK1 (n = 2); Figure S2. ^1^H NMR spectra of TK2 (n = 5); Figure S3. ^1^H NMR spectra of TK3 (n = 10); Figure S4. ^1^H NMR spectra of Phe-TK1-Phe (n = 2); Figure S5. ^1^H NMR spectra of Phe-TK2-Phe (n = 5); Figure S6. ^1^H NMR spectra of Phe-TK3-Phe (n = 10); Figure S7. ^1^H NMR spectra of gelator 1 (n = 2); Figure S8. ^1^H NMR spectra of gelator 2 (n = 5); Figure S9. ^1^H NMR spectra of gelator 3 (n = 10); Table S1: Critical gelation concentration and gelation behavior of three gelators in different solvents.
