# Supplementary material for: Constructing ROS-Responsive Supramolecular Gel with Innate Antibacterial Properties
Source: Pharmaceutics. 2023 Aug 19;15(8):2161. doi: 10.3390/pharmaceutics15082161 (PMC10458117; doi:10.3390/pharmaceutics15082161)
Supplement: Supplementary file 1 [file pharmaceutics-15-02161-s001.zip › pharmaceutics-2530164-supplementary.pdf]

pharmaceutics

# Supplementary Material: Constructing ROS-Responsive Supramolecular Gel with Innate Antibacterial Properties

**Fen Zheng, Wei Du, Minggang Yang, Kaige Liu, Shanming Zhang, Long Xu and Yong Wen**

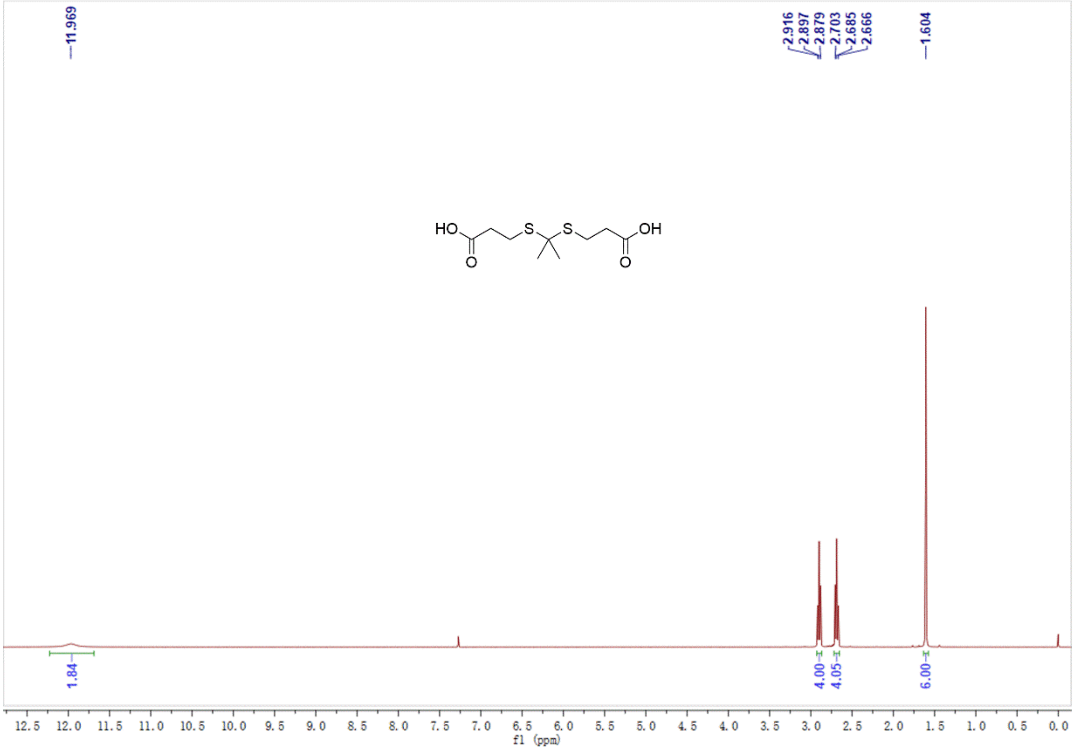


**Figure S1.** $^1$H NMR spectra of TK1 (n=2).

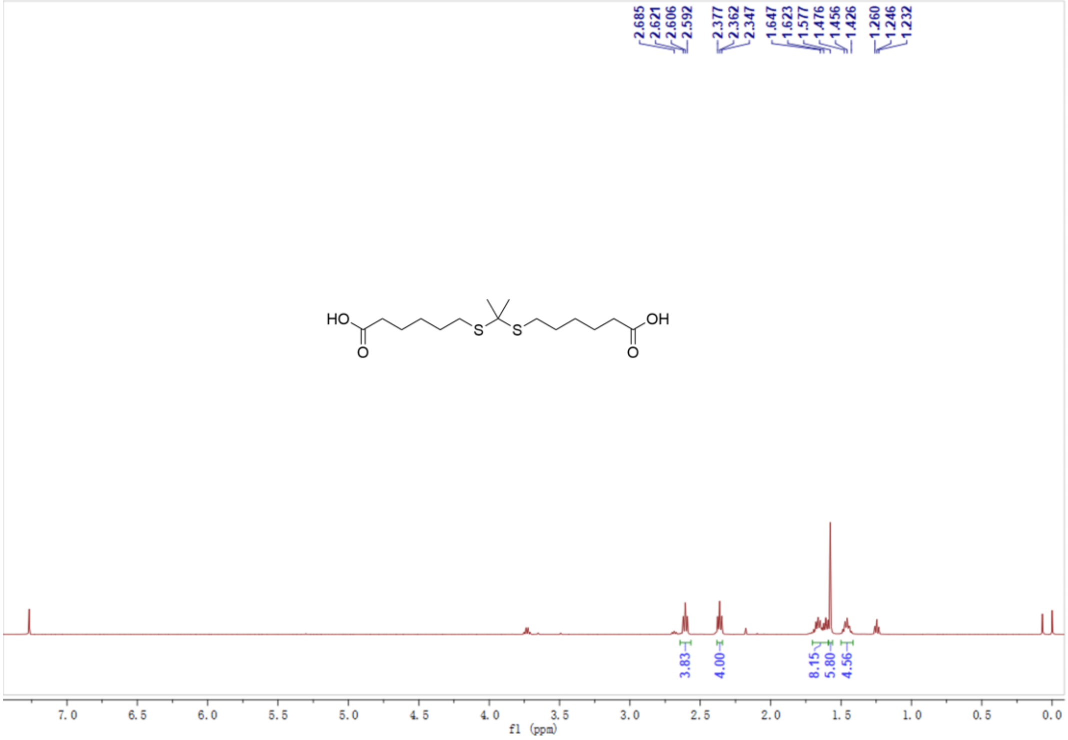


**Figure S2.** $^1$H NMR spectra of TK2 (n=5).

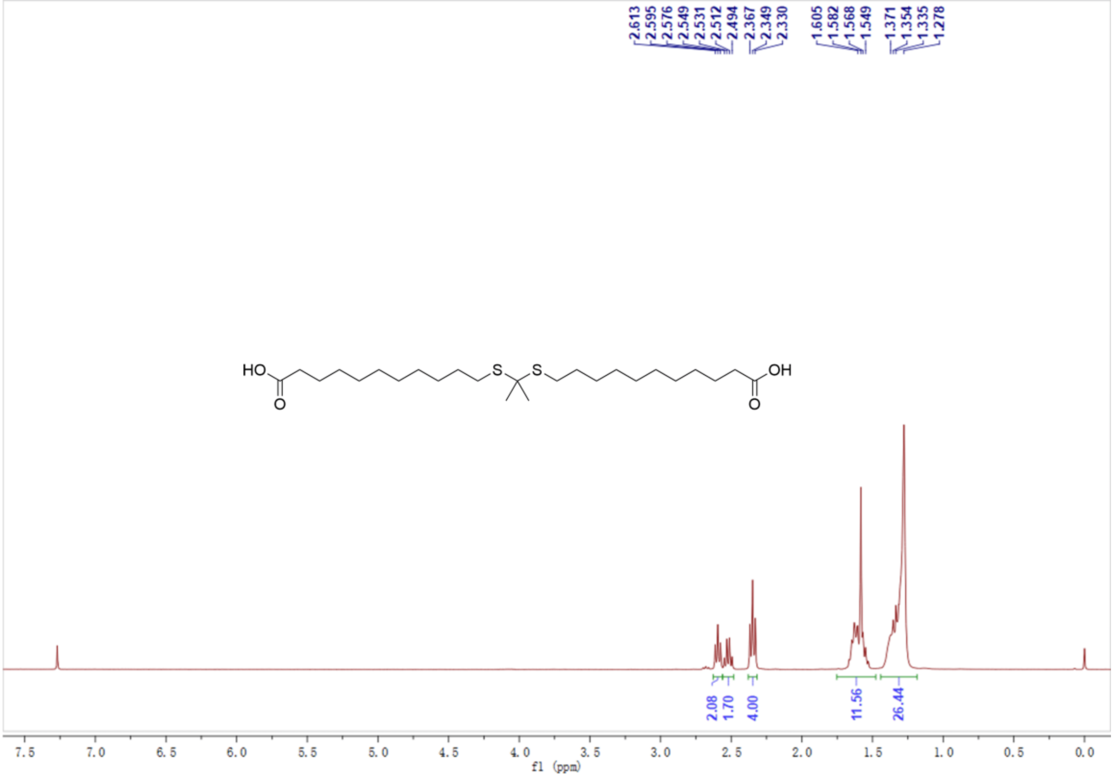

**Figure S3.** $^1$H NMR spectra of TK3 (n=10).

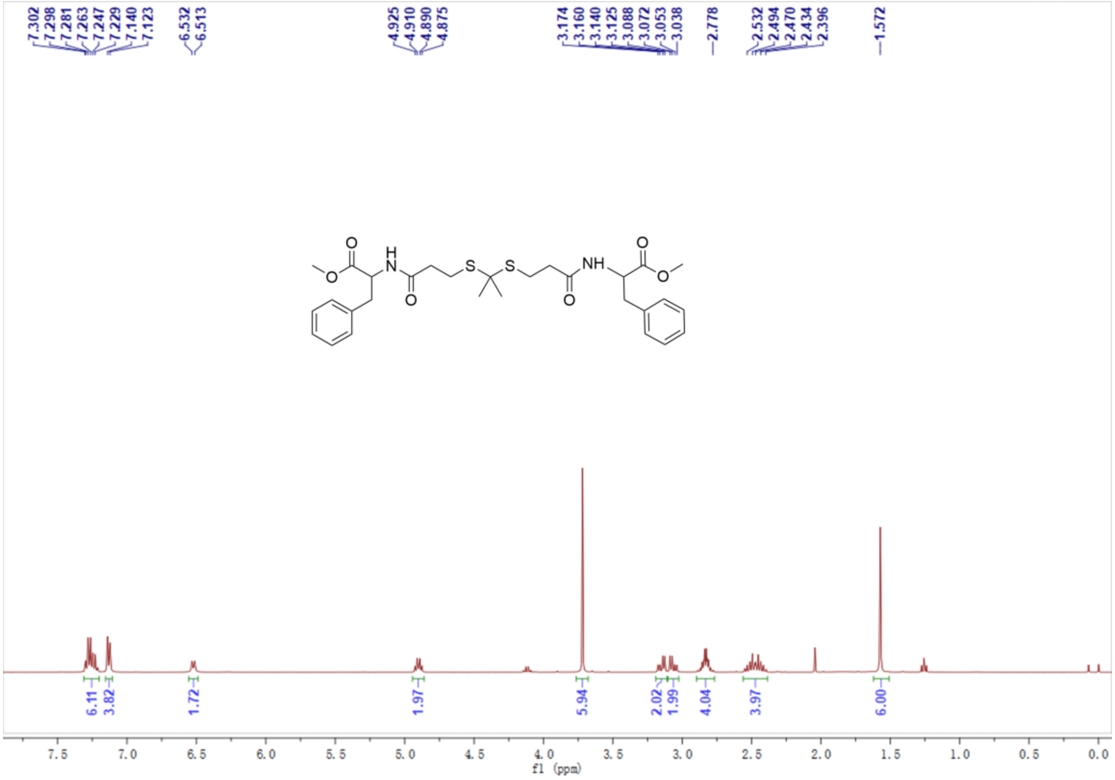

**Figure S4.** $^1$H NMR spectra of Phe-TK1-Phe (n=2).

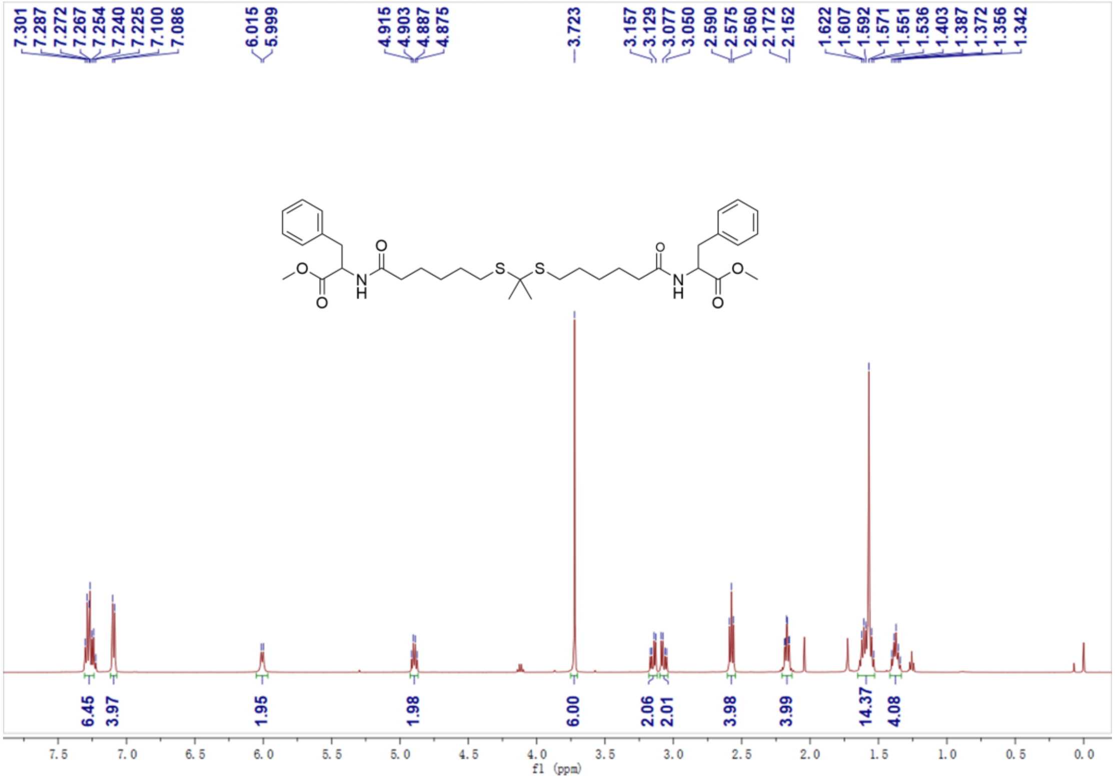


**Figure S5.** $^{1}$H NMR spectra of Phe-TK2-Phe (n=5).

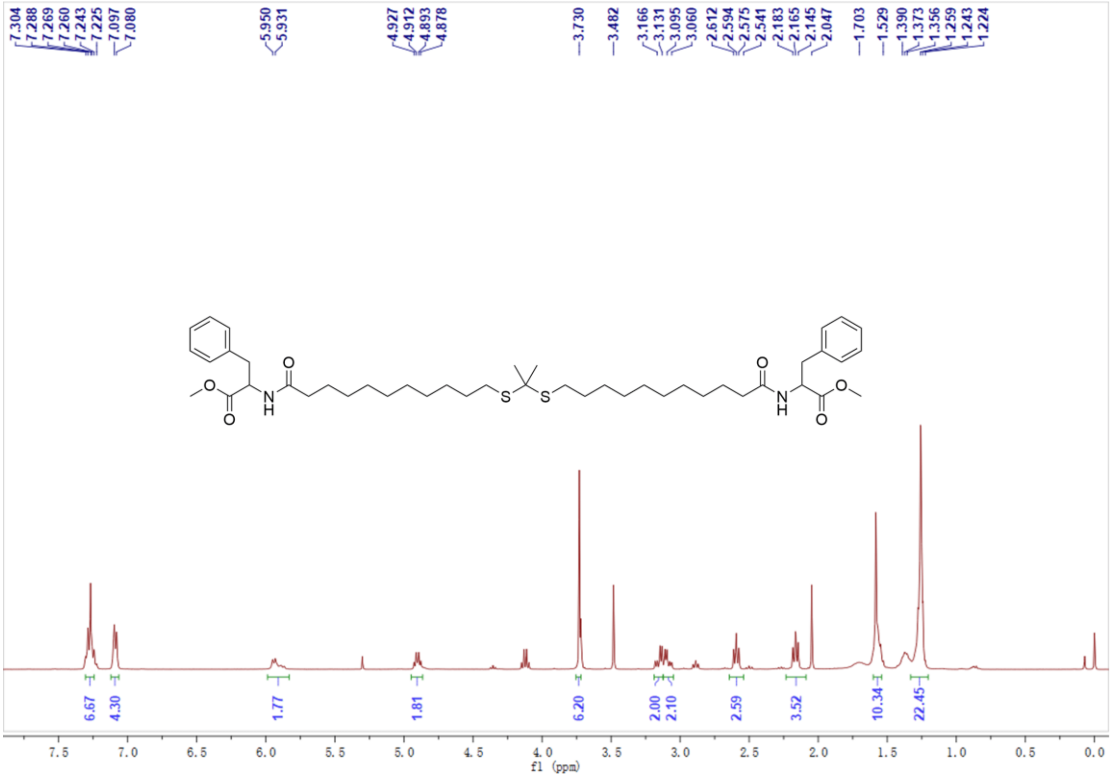


**Figure S6.** $^{1}$H NMR spectra of Phe-TK3-Phe (n=10).

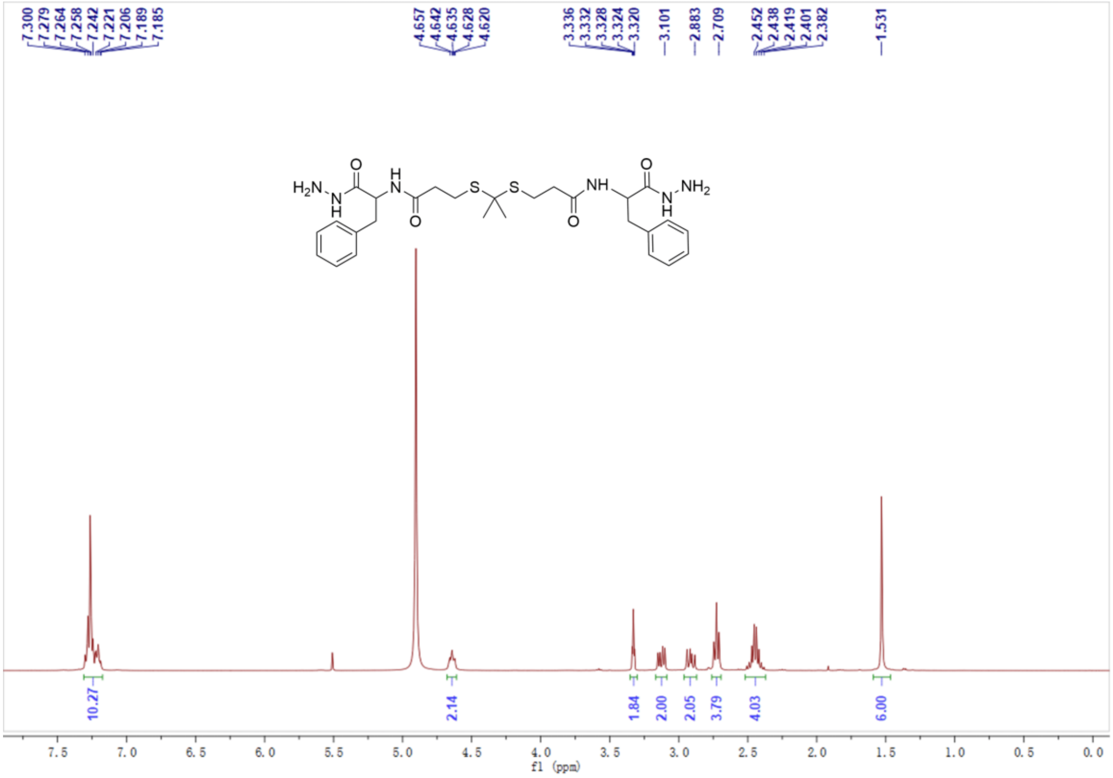

**Figure S7.** $^{1}H$ NMR spectra of gelator 1 (n=2).

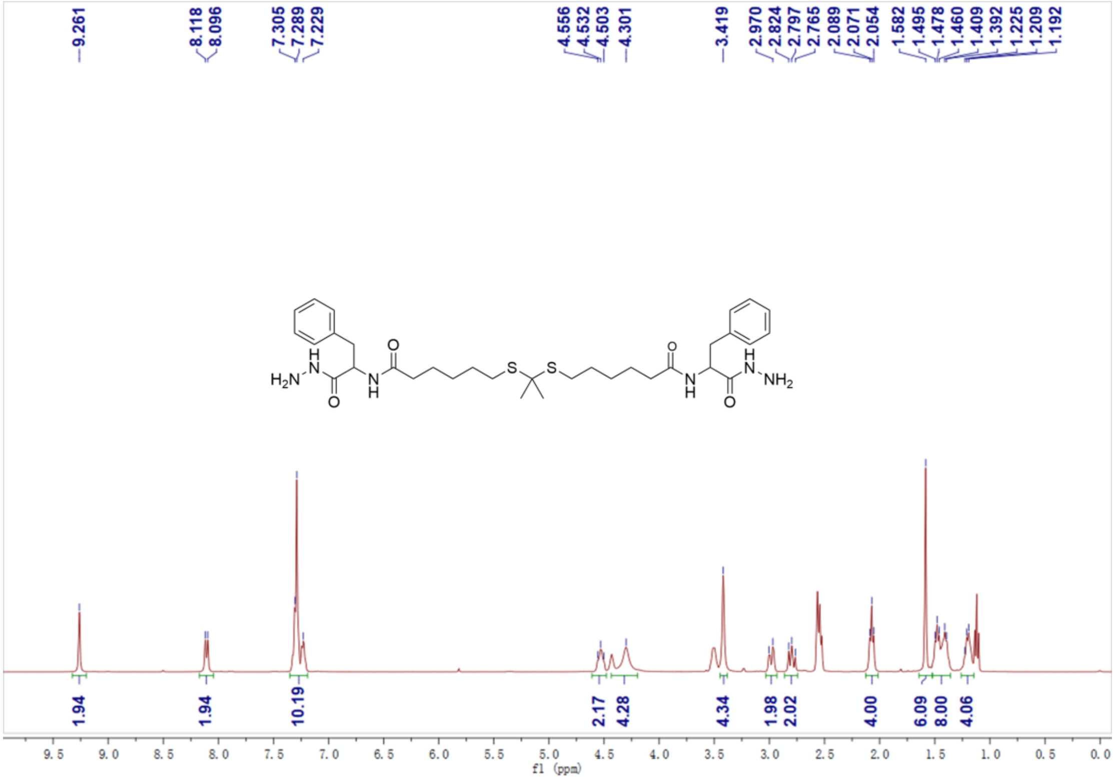

**Figure S8.** $^{1}H$ NMR spectra of gelator 2 (n=5).

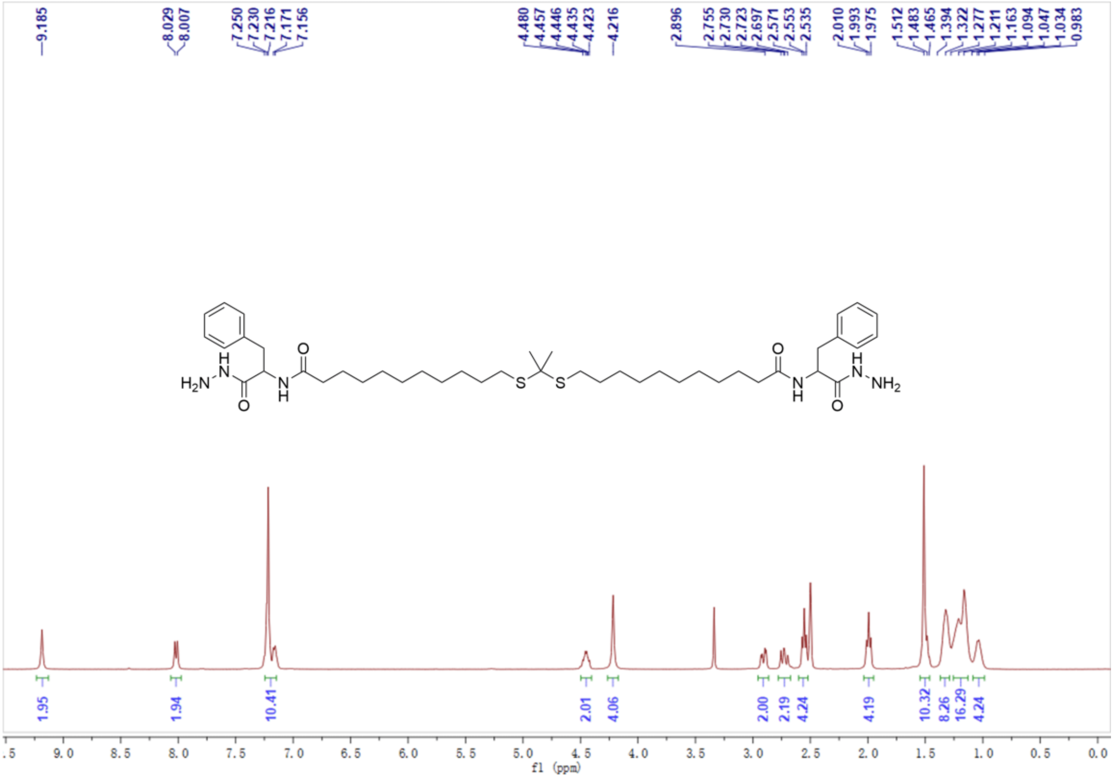


**Figure S9.** $^1$H NMR spectra of gelator 3 (n=10).

**Table S1.** Critical gelation concentration and gelation behavior of three gelators in different solvents.

| Entry | Organic solvent | Gelator 1 CGC at 25°C (mg/mL) | Gelator 2 CGC at 25°C (mg/mL) | Gelator 3 CGC at 25°C (mg/mL) |
|---|---|---|---|---|
| | Petroleum ether | I | I | I |
| 1 | Cyclohexane | I | I | I |
| 2 | Diethyl ether | I | I | I |
| 3 | Ethyl acetate | G (10 mg/mL) | G (6 mg/mL) | G (5 mg/mL) |
| 4 | Dichloromethane | G (10 mg/mL) | G (7 mg/mL) | G (3 mg/mL) |
| 5 | Chloroform | G (9 mg/mL) | G (10 mg/mL) | G (10 mg/mL) |
| 6 | Acetone | S | S | S |
| 7 | Acetonitrile | G (15 mg/mL) | G (5 mg/mL) | G (2 mg/mL) |
| 8 | Methanol | S | S | G (7 mg/mL) |
| 9 | Ethanol | S | G (18 mg/mL) | G (6 mg/mL) |
| 10 | Ethylene glycol | S | G (5 mg/mL) | G (12 mg/mL) |
| 11 | Dimethyl sulfoxide | S | S | S |
| 12 | PEG200 | S | S | G (15 mg/mL) |
| 13 | PEG400 | S | P | P |
| 14 | $H_2O$ | P | P | P |
| 15 | Ethanol : $H_2O$ (1:1) | I | G (10 mg/mL) | G (2 mg/mL) |
| 16 | PEG200 : $H_2O$ (1:1) | P | G (22 mg/mL) | G (18 mg/mL) |
| 17 | PEG200 : $H_2O$ (3:2) | P | G (28 mg/mL) | G (13 mg/mL) |
| 18 | | | | |

a) I: insoluble, if the gelator is completely insoluble in the solvent;
b) G: gel, if the gelator is able to gelation the solvent;
c) S: solution, if the gelator is completely soluble in the solvent;
d) P: precipitate, if the gelator dissolves after heating, and precipitates after cooling to room temperature.
